# Mobile Phone Apps to Improve Medication Adherence: A Systematic Stepwise Process to Identify High-Quality Apps

**DOI:** 10.2196/mhealth.6742

**Published:** 2016-12-02

**Authors:** Karla Santo, Sarah S Richtering, John Chalmers, Aravinda Thiagalingam, Clara K Chow, Julie Redfern

**Affiliations:** ^1^ The George Institute for Global Health University of Sydney Sydney Australia; ^2^ Sydney Medical School University of Sydney Sydney Australia; ^3^ Cardiology Department Westmead Hospital Sydney Australia; ^4^ Westmead Institute for Medical Research Sydney Australia; ^5^ Charles Perkins Centre Westmead Sydney Australia

**Keywords:** medication adherence, medication compliance, mobile phone, smartphone, mobile apps, mobile applications

## Abstract

**Background:**

There are a growing number of mobile phone apps available to support people in taking their medications and to improve medication adherence. However, little is known about how these apps differ in terms of features, quality, and effectiveness.

**Objective:**

We aimed to systematically review the medication reminder apps available in the Australian iTunes store and Google Play to assess their features and their quality in order to identify high-quality apps.

**Methods:**

This review was conducted in a similar manner to a systematic review by using a stepwise approach that included (1) a search strategy; (2) eligibility assessment; (3) app selection process through an initial screening of all retrieved apps and full app review of the included apps; (4) data extraction using a predefined set of features considered important or desirable in medication reminder apps; (5) analysis by classifying the apps as basic and advanced medication reminder apps and scoring and ranking them; and (6) a quality assessment by using the Mobile App Rating Scale (MARS), a reliable tool to assess mobile health apps.

**Results:**

We identified 272 medication reminder apps, of which 152 were found only in Google Play, 87 only in iTunes, and 33 in both app stores. Apps found in Google Play had more customer reviews, higher star ratings, and lower cost compared with apps in iTunes. Only 109 apps were available for free and 124 were recently updated in 2015 or 2016. Overall, the median number of features per app was 3.0 (interquartile range 4.0) and only 18 apps had ≥9 of the 17 desirable features. The most common features were flexible scheduling that was present in 56.3% (153/272) of the included apps, medication tracking history in 54.8% (149/272), snooze option in 34.9% (95/272), and visual aids in 32.4% (88/272). We classified 54.8% (149/272) of the included apps as advanced medication reminder apps and 45.2% (123/272) as basic medication reminder apps. The advanced apps had a higher number of features per app compared with the basic apps. Using the MARS instrument, we were able to identify high-quality apps that were rated as being very interesting and entertaining, highly interactive and customizable, intuitive, and easy to use and to navigate as well as having a high level of visual appeal and good-quality information.

**Conclusions:**

Many medication reminder apps are available in the app stores; however, the majority of them did not have many of the desirable features and were, therefore, considered low quality. Through a systematic stepwise process, we were able to identify high-quality apps to be tested in a future study that will provide evidence on the use of medication reminder apps to improve medication adherence.

## Introduction

Nonadherence to long-term therapies in chronic diseases is a global concern highlighted by the World Health Organization report in 2003 [1]. Medication nonadherence is associated with increased risk of morbidity [2], mortality [3], and costs [4]; therefore, there is a need for effective interventions to improve adherence. It is known that current interventions provide inconsistent results in improving adherence [5]. In recent years, the growing mobile phone ownership [6] has made mobile phones a promising tool to deliver health care interventions. Furthermore, there has been an increasing interest in using mobile phones as a tool to improve medication adherence.

Reminders sent via text messages have been shown to improve adherence in chronic diseases [7]. The recent growth in mobile phone subscriptions [6], however, has spawned an exponential increase in the number of health-related mobile phone apps available in the app stores, including those dedicated to improve medication-taking behavior. These medication adherence apps have many features, including reminders, that may help patients take their medication correctly and avoid medication errors and, hence, could address known barriers to adherence [8], especially for patients with high pill burden and complex regimens, such as patients with cardiovascular diseases.

Despite this plethora of medication adherence apps, there is a lack of information on how they differ, how many and which features they have, their overall quality, and whether they are effective. Previous reviews have identified available medication adherence–related apps and described the relevant features present in these apps [9-11]. However, these reviews only provided a descriptive analysis of the available apps and their features without a deeper quality assessment. The aim of this research was to describe a systematic and stepwise process to identify high-quality medication reminder apps by identifying and reviewing the current available apps and their features and to assess the apps’ quality by using a reliable quality assessment tool for mobile health apps.

## Methods

### Design

This review was conducted in a similar manner to a systematic review by using a stepwise approach that included a search strategy, prespecified eligibility criteria, app selection through an initial screening of all retrieved apps and full app review of the included apps, data extraction and analysis, and quality assessment of selected apps using a reliable quality assessment tool for mobile health apps.

### Search Strategy

The search was conducted in the main online app stores, iTunes (Apple Inc, Australia) and Google Play (Google Inc, Australia), that have more than 2 million apps available for download [12]. The apps available in these app stores are compatible with any mobile phone that uses the leading operating systems in Australia, iOS and Android systems, which together account for 97% of the Australian mobile phone market [13]. We, therefore, searched the Australian iTunes and Google Play app stores between December 10, 2015, and December 20, 2015, using 8 search terms that during the preliminary searches had the best performance in retrieving the apps of interest for this review. The search terms used were *medication reminder*, *medication pill reminder*, *pill reminder*, *meds reminder*, *medication tracker*, *medication management*, *Rx*, and *medication*.

### Eligibility Criteria

Apps were eligible to be included in this review if they met all the following inclusion criteria: (1) apps that aimed to support medication self-management, (2) apps capable of generating scheduled reminders for medication-taking behavior, and (3) apps that were in English.

As we aimed to include apps that could be used by a large number of patients or individuals, we excluded apps that were restricted to a specific group of individuals or type of medication, with the exception of apps related to cardiovascular diseases that have the highest prevalence worldwide. Therefore, apps were excluded from the review if they (1) generated general reminders not specifically designed for medication reminders (eg, general calendars and alarms); (2) focused on one medication (eg, contraception); (3) focused on individuals with only one medical condition, except cardiovascular diseases (eg, asthma); (4) focused on one specific group of people (eg, seniors); (5) only listed medication prescribed without sending reminders; (6) generated reminders for medication refill or expiration date without daily reminders for medication adherence; (7) were designed for ordering medication refills online (eg, pharmacy-owned apps); (8) were owned by health care services targeting only their own patients (eg, hospitals and family practices); (9) focused on general health, fitness, lifestyle, and well-being; (10) only provided medication information (eg, medication dosing information and side effects); and (11) lacked enough information to determine eligibility.

### App Selection Process

One author (KS) carried out the app store searches. Information about the apps retrieved from the search was entered into a predesigned electronic spreadsheet developed for this review. The information entered included name of the app, name of app developer, cost, app store or stores in which the app was available, and the search term or terms that retrieved the app. Apps available in both app stores were only entered once in the electronic spreadsheet, including same apps that had slightly different names or app developers’ name in the 2 app stores. Apps that had 2 versions in the same app store, for example, a free or lite version and a paid or pro version, were entered separately in the spreadsheet as the features may differ in each version.

All apps retrieved from the search were screened for eligibility by 1 reviewer (KS). The screening process consisted of reviewing the information available about the app characteristics on the product list in each app store, including written information, pictures, and videos. Additional apps found during the data extraction process were also added to the initial spreadsheet and screened for eligibility. Apps were included in this review if they met all the inclusion criteria, and the reasons for exclusion were recorded. A full detailed review of the apps included in this review was performed.

### Data Extraction and Analysis

Before data extraction, a set of features considered important or desirable in medication reminder apps was developed for this review based on previous reviews [9-11] and on a panel consensus (KS, CKC, and JR). The set of important features consisted of 3 practical and 17 functionality features (Table 1). To assess the features present in the apps, the information available about the app from the app store, including the written description, photos, and videos, was extracted. If an app was available in both app stores, the information available in both app stores was combined for the review. In addition, information about the app or the app developer provided on its own website was also extracted and assessed. The information extracted included name of the app, name of the app developer, app store in which the app was available, star rating, number of reviews, cost, and the last date updated, as well as the practical and functionality features. For each practical and functionality feature, the reviewer determined if the criteria were present or absent. If the presence of a feature could not be ascertained by evaluating the different sources of information described above, the feature was considered to be absent in the app.

**Table 1:** Practical and functionality features’ description.

Features		Rationale or description
**Practical features**		
	Available in both app stores	Allows the app to be used by individuals who own mobile phones that use the leading operating systems (iOS and Android).
	Available for free without ads	A full free version of the app without advertising for third-party products is likely to be used by a large number of individuals.
	Updated in 2015 or 2016	A recent update ensures ongoing technical support to fix any software issues.
**Functionality features**		
	Medication tracking history	Ability to record and track taken and missed doses.
	Snooze option	Ability to snooze the reminder for a predetermined period of time, for example, 15 minutes.
	Flexible scheduling	Ability to schedule reminders to occur on a nondaily or monthly basis or every X days, or ability to schedule medications with stop dates.
	Medication database	Availability of a medication database that allows the user to search and select a medication from the database.
	Refill reminders	Ability to set reminders to the date when medication needs to be refilled.
	Reminders with no connectivity	No Internet connection required for the reminders to function.
	Data exporting and sharing	Ability to export and share the medication information to a third party, for example, family member or health care provider.
	Multiple users support	Ability to generate medication reminders on different medications for more than 1 user, for example, family members.
	Notification for other people	Availability of an option to alert other people about when to take their medication or when missed doses are registered.
	Data security	The app developer ensures data security, for example, data are only stored in the user’s device or statement of HIPAA^a^ compliance.
	Data privacy: password protection	Password option to access the app.
	Multilingual	Availability of languages other than English.
	Time zone support	Ability to change time zones to ensure medication is taken at the right time when traveling.
	Adherence rewards	Availability of a feature that rewards the patient when the medication is taken on schedule, for example, awarding points for each medication taken that could be redeemed into vouchers.
	Adherence statistics and charts	Availability of statistics and charts describing medication usage trends and adherence rates.
	Customizable alert sounds	Availability of different types of notification sounds.
	Visual aids	Availability of icons (eg, tablet, syringe, drops) or ability to add a picture to provide visual clues (eg, to ensure correct medication is taken).

^a^HIPAA: Health Insurance Portability and Accountability Act.

Extracted data were entered into an electronic spreadsheet and were analyzed using IBM SPSS version 22.0 (IBM Corporation). App characteristics and features were summarized as means or medians for continuous data and as frequencies and proportions for categorical data. We further analyzed the included apps by (1) classifying them as basic or advanced medication reminder apps and (2) ranking the apps using a scoring system developed for this review.

#### Classification of Apps

On the basis of the presence or absence of the medication tracking history feature, each app was classified into the following categories that were previously described by Stawarz et al [10]: (1) basic medication reminder apps and (2) advanced medication reminder apps.

The first type of app offers basic features to support prospective memory, by providing daily, simple, timed reminders to reinforce medication-taking behavior with no further interactivity. This basic app acts similarly to an alarm or a text message by showing a reminder on the mobile phone screen at a set time every day.

The second type of app offers not only the same basic features to support prospective memory, by providing the same daily, simple, timed reminders to reinforce medication-taking behavior, but also additional features to support retrospective memory, by having the ability to track the medications taken or missed, as well as having more customizable and interactive features, such as adherence statistics, time zone support, data sharing, and multiple user support.

#### App Ranking

The apps in each of the categories described above were ranked based on a scoring system developed for this review by assessing the number of practical and functionality features detailed in Table 1. To rank the apps, we calculated a score for each app by adding 2 points for each practical feature present in the app and 1 point for each functionality feature. The practical features were given a higher weight in the scoring system as these features were considered important to ensure that the apps are available for a large number of people. The total number of points possible was therefore 23, where a maximum of 6 points were available for practical features and a maximum of 17 points were available for functionality features. The apps were then ranked from highest to lowest, where those with the most practical and functional features were ranked highest.

### Quality Assessment Using the Mobile App Rating Scale

As it was not feasible to download all the apps included in this review, we decided to select the top 5 scoring apps in each category (basic and advanced medication reminder apps) for further assessment using the Mobile App Rating Scale (MARS) [14]. The MARS tool is a 23-item scale developed by researchers to assess the quality of mobile health apps. The MARS instrument provides a deeper evaluation of the app quality by testing the app thoroughly for 10 minutes and grading the app in several domains, including user engagement, functionality, aesthetics, information, and app subjective quality. Each item was scored using a 5-point scale (1-Inadequate, 2-Poor, 3-Acceptable, 4-Good, 5-Excellent). For each domain, we calculated a mean score that ranged from 0 to 1, where a score of 0 would mean inadequate quality and a score of 1 would mean excellent quality.

In the selection process for app download, if more than 5 apps had the same scores, the apps were selected for download using the following predefined hierarchy: apps available in both app stores, apps for free, and apps updated in 2015 or 2016. Two reviewers (KS and SR) downloaded and independently tested the selected apps using the MARS instrument. The reviewers were trained to use the MARS instrument by watching an online tutorial to ensure that both reviewers used the tool in the same manner. One reviewer (KS) assessed the apps using an iOS device and the other reviewer (SR) used an Android device. In addition, the reviewers were required to check if all the functionality features described in the app store were, in fact, present in the app. Any disagreement was discussed and resolved by consensus.

## Results

### Search

**Figure 1:**
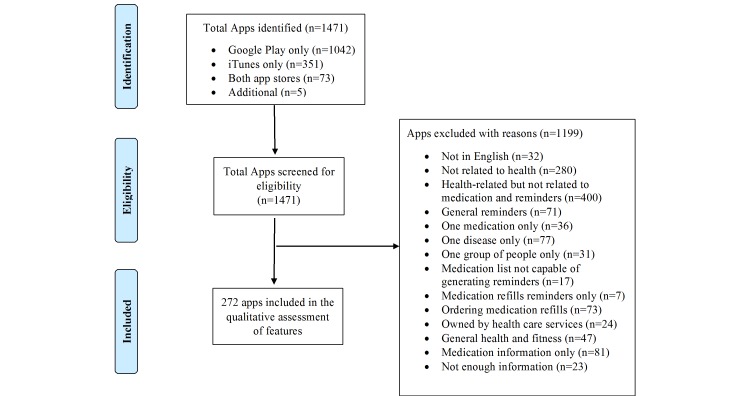
Flowchart of selection of included apps.

The process of identification and inclusion of apps is outlined in Figure 1. A total of 1471 apps were screened for eligibility and 1199 apps were excluded for several reasons presented in Figure 1. A total of 1042 apps were found only in Google Play, 351 only in iTunes, and 73 in both app stores. Another 5 apps were identified during the data extraction process and were also screened for eligibility. After exclusions, 272 apps were eventually included in this review.

### General Characteristics of Included Apps

Of the 272 apps included, 55.9% (152/272) were found only in Google Play, 32.0% (87/272) only in iTunes, and 12.1% (33/272) in both app stores. A total of 34 apps had 2 versions: a free or lite version and a paid or pro version. In Google Play, 91.9% (170/185) of the apps were reviewed by customers who gave a star rating to the apps (star ratings range from 0 to 5 stars). The median star rating for Google Play apps was 3.9 (interquartile range, IQR, 0.82) with a minimum of 1.0 star given to 2 apps and a maximum of 5.0 stars given to 17 apps. The median number of reviews per app was 22.5 (IQR 78.75), with a minimum of 1 review for 12 apps and a maximum of 98,179 reviews for 1 app (Medisafe app). In terms of cost, 20.5% (38/185) of the apps found in Google Play required a payment for download at a median cost of Aus $1.88 (IQR 1.63) and a range of Aus $0.99 to Aus $5.70.

In the iTunes store, only 34.2% (41/120) of the apps were reviewed by customers who gave a star rating to the apps (star ratings range from 0 to 5 stars). The median star rating for iTunes apps was 3.0 (IQR 2.75) with a minimum of 1.0 star given to 9 apps and a maximum of 5.0 stars given to 5 apps. The median number of reviews per app was 2.0 (IQR 9.5), with a minimum of 1 review for 11 apps and a maximum of 153 reviews for 1 app (Medisafe app). In terms of cost, 48.3% (58/120) of the apps found in iTunes required a payment for download at a median cost of Aus $2.99 (IQR 3.00) and a range of Aus $1.49 to Aus $42.99.

### Features of Included Apps

**Figure 2:**
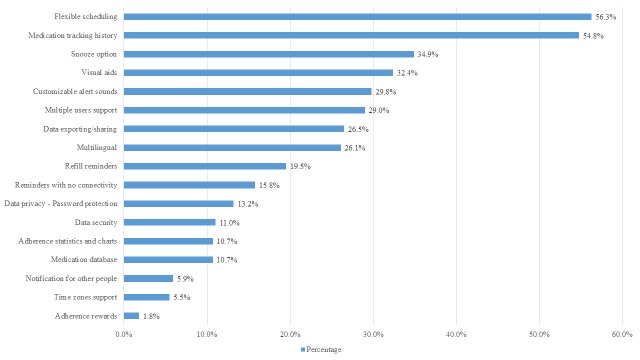
Results of functionality criteria assessment.

In terms of the practical features, as stated above, 12.1% (33/272) of the apps were available in both app stores. In addition, 40.1% (109/272) were fully available for free without third-party advertisement or in-app purchases and 45.6% (124/272) were recently updated in 2015 or 2016. In terms of functionality, the median number of features per app was 3.0 (IQR 4.0) and only 6.6% (18/272) of the apps had at least 9 of the 17 desirable features. Flexible scheduling and medication tracking history were the only 2 features present in more than half of the apps. Other common functionality features were snooze option, visual aids, customizable alert sounds, multiple users support, data exporting and sharing, and languages other than English, which were present in around a third of the apps. All the other functionality features were present in less than a quarter of the apps (Figure 2).

### Classification and Ranking of Included Apps

In total, 54.8% (149/272) of the included apps were classified as advanced medication reminder apps as they had the ability to track taken and missed doses, while the other 45.2% (123/272) were classified as basic medication reminder apps. In terms of functionality features according to the classification group, the advanced apps had more than double the number of features compared with the basic apps, having a median of 5 (IQR 4) and 2 (IQR 2) features per app, respectively. Among the advanced apps, the apps with the highest number of functionality features were Medisafe and Pill Reminder by Drugs.com with 14 features, while among the basic apps it was AlarMeds reminder app with 7 features.

Regarding the ranking of the apps, Medisafe was ranked number 1 among the advanced medication reminder apps, achieving 20 out of a maximum of 23 points (Table 2). The median score among the advanced apps was 7 (IQR 5), having a score range of 1 to 20 points. Among the basic medication reminder apps, My heart, my life was the highest-scoring app, achieving 9 points. The median app score in this category was 4 (IQR 3), having a score range of 0 to 9. The practical and functionality features in the top-ranking apps among the advanced and basic medication adherence apps are presented in Tables 2 and 3.

**Table 2:** Rank and score of and practical features present in the top advanced and basic medication reminder apps.

Rank	App names	Score	Available in both app stores	Full version available for free	Updated in 2015 or 2016
**Advanced apps**					
1	Medisafe	20	✓^a^	✓	✓
2	Dosecast	15	✓		✓
3	MyMeds	15	✓		✓
4	CareZone	14	✓	✓	✓
5	My Pillbox	14	✓		✓
6	MedicineList+^b^	14	✓	✓	✓
**Basic apps**					
1	My heart, my life	9	✓	✓	✓
2	MediWare^c^	8	✓	✓	✓
3	MyMedManager	8	✓	✓	
4	Pill Reminder (Aplicativos Legais)^d^	8	✓		✓

^a^The symbol ✓ means that the feature was present in the app when tested in both iOS and Android devices.

^b^Initially evaluated as a basic medication reminder app but after download classified as an advanced app as the medication tracking history feature was present.

^c^Could not be assessed owing to crashes and technical issues in both iOS and Android devices.

^d^Only assessed on an iOS device because of technical problems in the Android device.

**Table 3:** Functionality features present in the top advanced and basic medication reminder apps.

App names		Functionality features^a^																
		1	2	3	4	5	6	7	8	9	10	11	12	13	14	15	16	17
**Advanced apps**																		
	Medisafe	✓^b^	✓	✓	✓	✓	✓	✓	✓	✓			✓	✓		✓	✓	✓
	Dosecast	✓	✓	✓		✓	✓	✓	✓		✓						✓	✓
	MyMeds	✓	✓		✓	✓	✓		✓		✓	✓			✓	✓		✓
	CareZone	✓	✓	✓	✓	✓	✓	✓	✓	✓	✓			✓		✓		✓
	My Pillbox	✓	✓	✓		✓	✓	✓	✓			✓					✓	✓
	MedicineList+^c^	✓	✓	✓	✓		✓	✓	✓		✓							
**Basic apps**																		
	My heart, my life			✓	✓						✓	✓						✓
	MediWare^d^																	
	MyMedManager		✓	✓			✓										✓	
	Pill Reminder (Aplicativos Legais)^e^																	

^a^See Table 1 for the 17 functionality features.

^b^The symbol ✓ means that the feature was present in the app when tested in both iOS and Android devices.

^c^Initially evaluated as a basic medication reminder app but after download classified as an advanced app as the medication tracking history feature was present.

^d^Could not be assessed owing to crashes and technical issues in both iOS and Android devices.

^e^Only assessed on an iOS device because of technical problems in the Android device.

### Quality Assessment Using the Mobile App Rating Scale

Medisafe was the highest-scoring app of the 10 apps assessed using the MARS instrument; as it had the highest scores in the engagement and aesthetics domains because it was found to be interesting, entertaining, highly interactive, and customizable and to have a high level of visual appeal (Table 4). In addition, Medisafe had the maximum score in the subjective quality section, meaning that the reviewers would use this app regularly and recommend it to others. Medisafe was also the only app rated as having some evidence supporting its effectiveness in nonrandomized studies. The My heart, my life app had the maximum score in the functionality domain as it was intuitive, was easy to use and to navigate, and did not present any technical issues during use. The MedicineList+ app had the highest score in the information domain as it provided high-quality information from a credible source called National Prescribing Service MedicineWise Australia, which is an independent not-for-profit organization.

**Table 4:** The Mobile App Rating Scale mean scores assessed by domains.

App names		Mean scores by domains^a^					
		Engagement	Functionality	Aesthetics	Information	Subjective quality	MARS^b^ total score
**Advanced apps**							
	Medisafe	0.94	0.90	0.93	0.83	1.00	0.92
	MedicineList+^c^	0.74	0.90	0.87	0.93	0.78	0.84
	CareZone	0.78	0.90	0.93	0.84	0.70	0.83
	My Pillbox	0.76	0.83	0.77	0.68	0.53	0.71
	Dosecast	0.56	0.90	0.80	0.80	0.55	0.70
	MyMeds	0.52	0.75	0.87	0.55	0.48	0.63
**Basic apps**							
	My heart, my life	0.60	1.00	0.83	0.82	0.58	0.77
	MyMedManager	0.50	0.83	0.70	0.63	0.33	0.60
	Pill Reminder (Aplicativos Legais)^d^	0.36	0.80	0.60	0.33	0.25	0.47
	MediWare^e^	0.00	0.00	0.00	0.00	0.00	0.00

^a^Mean score ranges from 0 to 1, where a score of 0 means inadequate quality and a score of 5 means excellent quality.

^b^MARS: Mobile App Rating Scale.

^c^Initially evaluated as a basic medication reminder app but after download classified as an advanced app as the medication tracking history feature was present.

^d^Only assessed on an iOS device because of technical problems in the Android device.

^e^Could not be assessed owing to crashes and technical issues in both iOS and Android devices.

On the basis of the MARS assessment, Medisafe app was evaluated as the best app currently available in the app stores overall and among the advanced medication reminder apps, while My heart, my life was the best available app among the basic medication reminder apps. MedicineList+ app was classified as a basic medication reminder app in the initial assessment of features during the data extraction process; however, after the app was downloaded for quality assessment using the MARS tool, it was reclassified as an advanced app as this app had the ability to record and track taken and missed doses.

## Discussion

### Principal Findings

This review documents a systematic stepwise process to identify high-quality medication reminder apps. A comprehensive search identified 272 medication reminder apps, of which only a small number of apps were available in both app stores. Importantly, less than half of the apps were fully available at no cost and have been recently updated. In addition, the average number of desirable features per app was low and only a very small number of apps had more than half of these important features, and, therefore, the majority of apps were considered low quality. About half of them were classified as advanced medication reminder apps, while the other half was classified as basic apps. As expected, advanced apps had a higher average number of features and higher scores compared with basic apps. Through an in-depth quality assessment using a reliable tool, high-quality medication reminder apps were identified.

Previous reviews have also attempted to evaluate the availability of apps related to medication adherence in the app stores and their features. Similar to our results, other authors have found a large number of medication reminder apps available in the app stores with only a small number being available in more than one app store [9-11,15]. In addition, Bailey et al [11] also found that approximately half of the apps had a medication tracking history feature. However, these reviews only provided a descriptive analysis of the available apps and their features without a deeper quality assessment. Furthermore, these reviews are dated and were performed using different app stores in the United States and United Kingdom. It is important to mention that the app stores in different countries have different apps available, as the choice of countries in which the apps are available is determined by the app developers [16,17]. In this review, among the 10 apps selected for download, 2 apps (My heart, my life and MedicineList+) are available only in Australia as they were developed by Australian not-for-profit organizations.

It is important to highlight that the current mobile health app market is poorly regulated. The app stores provide guidelines about restricted content, privacy and security of the data, and monetization of the apps; however, these guidelines are not a quality control assessment of the available apps. Recently, the US Food and Drug Administration (FDA) released a guidance document stating which type of mobile medical apps will be subject to their regulation [18]. However, at this stage, apps to promote medication adherence will not be within the FDA regulation oversight, as these apps are not intended to provide diagnosis or treatment recommendations.

The lack of quality control and assessment makes it difficult for individuals to choose and even for health professionals to recommend high-quality apps to their patients. In this work, the described process to identify high-quality apps might be useful to guide others researchers, clinicians, and stakeholders on how to assess mobile health apps. First, a systematic and comprehensive search of the app stores is needed to ensure that all available apps are identified. Second, inclusion and exclusion criteria should be predefined to select apps that are appropriate for a target population. Third, the features that are considered essential and desirable to the apps being evaluated should be predetermined so that higher-quality apps can be identified. Finally, download and testing of selected apps using a quality assessment tool can confirm whether these apps are of high quality.

This review is not without limitations. The search conducted in the Australian app stores might have restricted the results of this review as some of the selected apps are only found in Australia. However, one of the aims of this review was to detail a process for use by future researchers who might need to identify and assess apps in their region and area of interest. In addition, the rigorous eligibility criteria might have resulted in the exclusion of some good-quality medication reminder apps that might be suitable for specific groups of patients. We also acknowledge our inability to download and assess all the included apps; however, we believe that this limitation did not compromise our results. Although we were able to identify high-quality medication reminder apps, currently there is no evidence that these apps are effective in improving medication adherence. To fill this gap in knowledge, our team is designing a study (including a qualitative component) to test the high-quality apps identified in this review. If proven effective, medication reminder apps can have an impact on clinical practice as they can be used as an additional tool among other strategies to improve adherence.

### Conclusions

In the current technology-driven world, apps have been gaining space in our everyday lives. Health apps, including medication reminder apps, are becoming more and more popular and are a promising tool to improve people’s health. In this review, we found a large number of medication reminder apps available in the app stores; however, majority of them were considered low quality. Through a systematic stepwise process, we were able to identify high-quality apps to be tested in a future study that will provide evidence on the use of medication reminder apps to improve medication adherence.

## Abbreviations

FDA: US Food and Drug Administration
IQR: interquartile range
MARS: Mobile App Rating Scale
